# Novel *de novo* missense mutation in the interferon regulatory factor 6 gene in an Italian infant with IRF6-related disorder

**DOI:** 10.1186/s13052-022-01330-6

**Published:** 2022-07-29

**Authors:** Ingrid Anne Mandy Schierz, Salvatore Amoroso, Vincenzo Antona, Mario Giuffrè, Ettore Piro, Gregorio Serra, Giovanni Corsello

**Affiliations:** 1 Neonatal Intensive Care Unit, Department of Health Promotion, Mother-Child Care, Internal Medicine and Medical Specialties “G. D’Alessandro”, University Hospital “P. Giaccone”, Via Alfonso Giordano n. 3, 90127 Palermo, Italy; 2 Pediatric Surgery Unit, Children’s Hospital, ARNAS Civico - Di Cristina - Benfratelli, Palermo, Italy

**Keywords:** Case report, Syngnathia, Orofacial cleft, Ankylosis, Syndactyly, Popliteal pterygium syndrome, Van der Woude syndrome, *IRF6*

## Abstract

**Background:**

Congenital maxillomandibular syngnathia is a rare craniofacial anomaly leading to difficulties in feeding, breathing and ability to thrive. The fusion may consist of soft tissue union (synechiae) to hard tissue union. Isolated cases of maxillomandibular fusion are extremely rare, it is most often syndromic in etiology.

**Case presentation:**

Clinical management of a female newborn with oromaxillofacial abnormities (synechiae, cleft palate, craniofacial dysmorphisms, dental anomaly) and extraoral malformations (skinfold overlying the nails of both halluces, syndactyly, abnormal external genitalia) is presented. The associated malformations addressed to molecular genetic investigations revealing an interferon regulatory factor 6 (IRF6)-related disorder (van der Woude syndrome/popliteal pterygium syndrome). A novel *de novo* heterozygous mutation in exon 4 of *IRF6* gene on chromosome 1q32.2, precisely c.262A > G (p.Asn88Asp), was found. Similarities are discussed with known asparagine missense mutations in the same codon, which may alter *IRF6* gene function by reduced DNA-binding ability. A concomitant maternal Xp11.22 duplication involving two microRNA genes could contribute to possible epigenetic effects.

**Conclusions:**

Our reported case carrying a novel mutation can contribute to expand understandings of molecular mechanisms underlying synechiae and orofacial clefting and to correct diagnosing of incomplete or overlapping features in IRF6-related disorders. Additional multidisciplinary evaluations to establish the phenotypical extent of the IRF6-related disorder and to address family counseling should not only be focused on the surgical corrections of syngnathia and cleft palate, but also involve comprehensive otolaryngologic, audiologic, logopedic, dental, orthopedic, urological and psychological evaluations.

## Background

Congenital maxillomandibular syngnathia (fusion) is a rare craniofacial anomaly leading to difficulties in feeding, breathing and ability to thrive. Isolated cases of syngnathia are extremely rare, it is most often syndromic in etiology. The presentation may be midline (anterior), unilateral or bilateral, partial or complete [1]. The entity of fusions can also vary from soft tissue unions (synechiae) to hard tissue unions (synostoses). Historically, the first description of a bony fusion of unknown etiology dates back to 1871 by Bohemian anatomist Bochdalek [2], while the first ascertained congenital syngnathia is from 1907 [1]. To date, the true prevalence is still unknown. On the one hand, this seems to be due to inconsistent nomenclature and classification systems, and, on the other hand, to the fact that only singular case reports are described. However, syngnathia is likely to be underreported, since milder isolated soft tissue bands in wellbeing newborns could often be undetected. Moreover, fusions in the context of polymalformative syndromes could not always be specified as a single entity. If the treatment is delayed, underdevelopment of mandible as well as extra-articular ankylosis of the temporomandibular joint may result [3].

We report on a female newborn with multiple bilateral maxillomandibular fibrous bands. The associated malformations addressed to molecular genetic investigations revealing an interferon regulatory factor 6 (IRF6)-related disorder. She presents a novel *de novo* heterozygous mutation in exon 4 of *IRF6* gene on chromosome 1q32.2, precisely c.262A > G (p.Asn88Asp).

## Case presentation

**Fig. 1:**
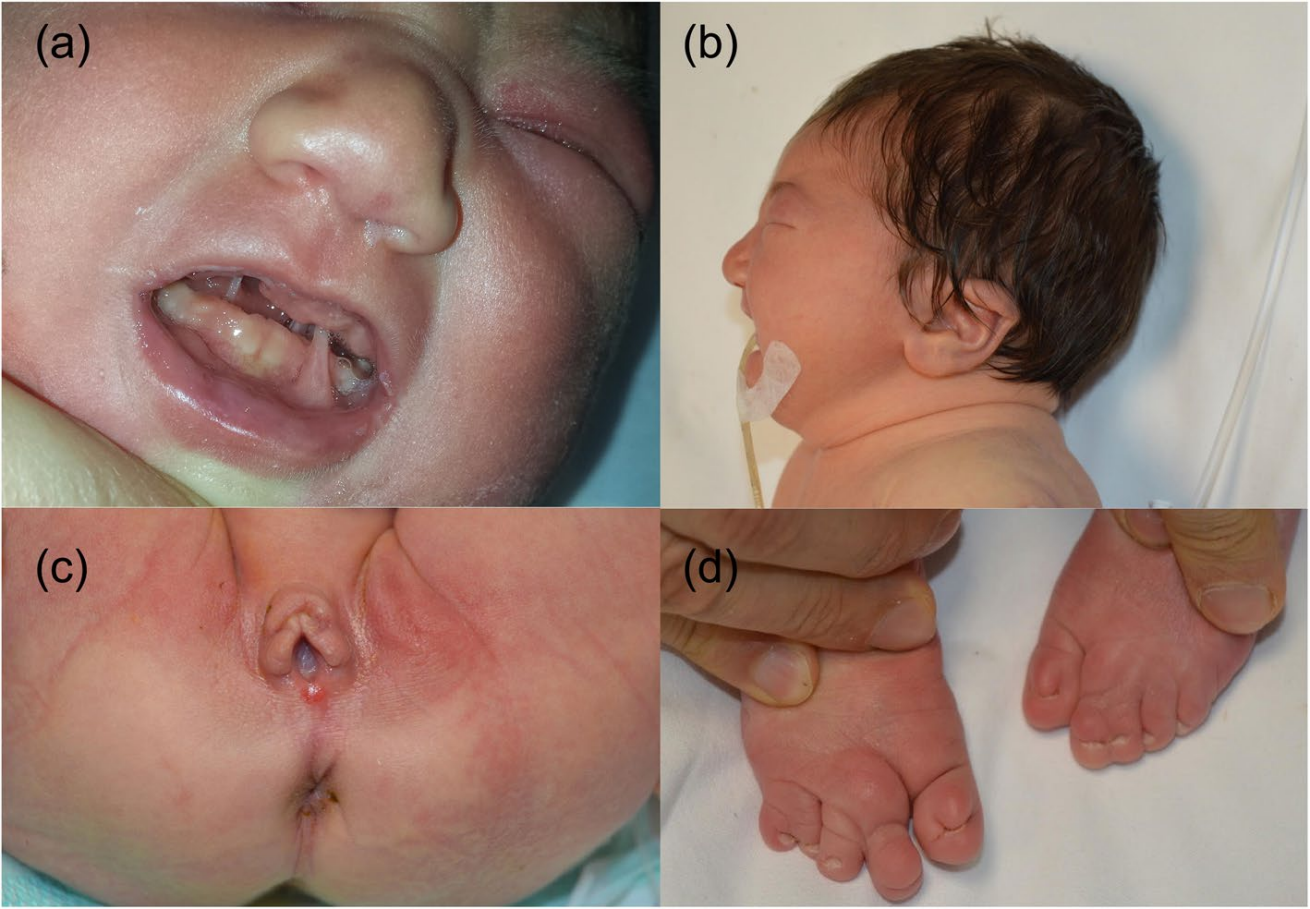
Characteristic features of the IRF6-related disorder at birth. **a** Broad nasal bridge, thin lips, mild retrognathia and numerous oral synechiae. **b** Lateral view of low-set malformed ears. **c** Hypertrophy of clitoris and hypoplasia of the labia majora. **d** Bilateral cutaneous syndactyly of II and III toes, cutaneous syndactyly of IV and V right toes, excessive subcutaneous tissue on the dorsum of the II right toe (resembling a plantar aspect), and pyramidal skinfold overlying the nails of both halluces

**Fig. 2:**
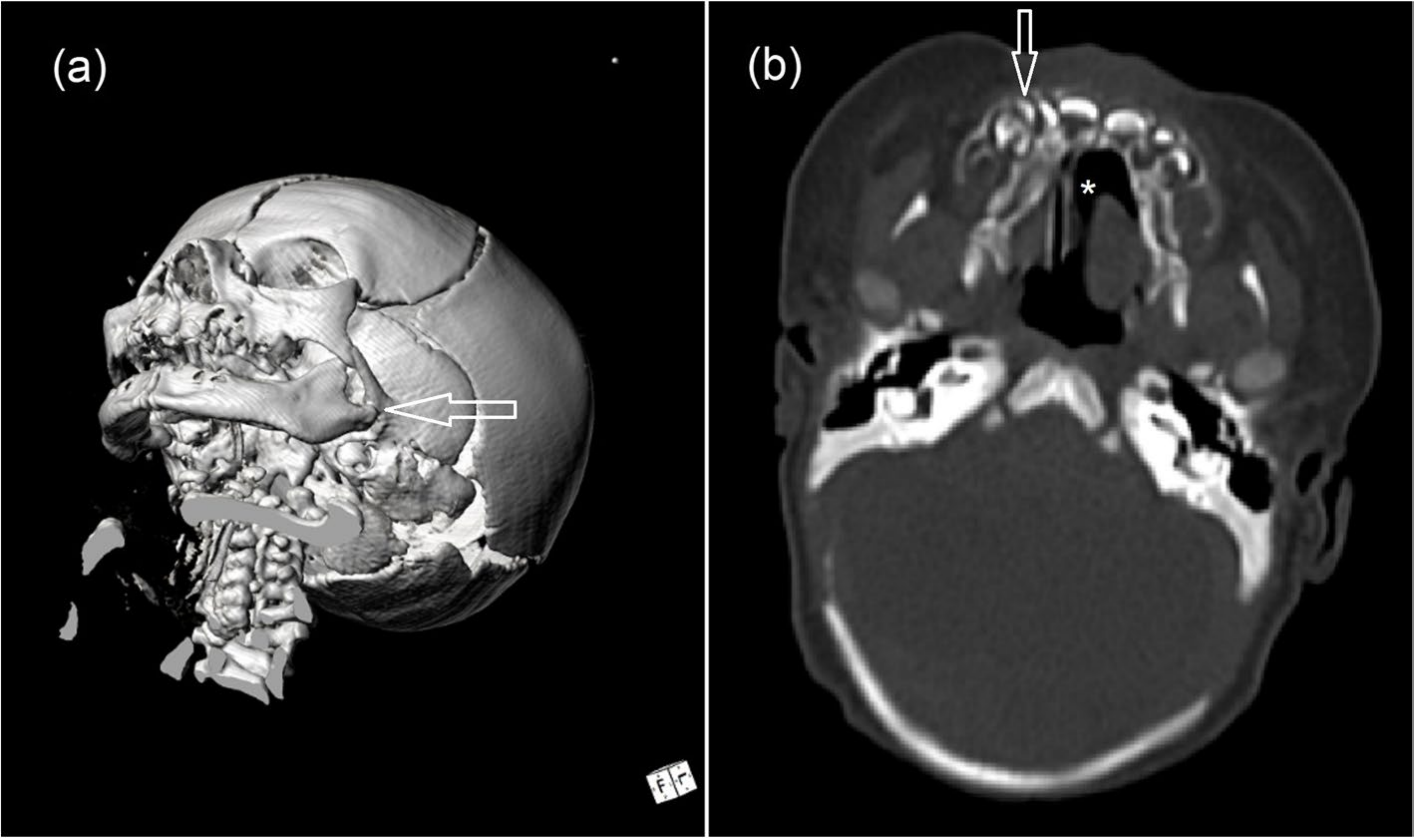
**a** Presurgical 3D reconstruction shows underdeveloped mandibular condyles, reduced temporomandibular joint spaces, flattening of bone components (arrow), but no bony fusions. **b** Tomographic axial view shows cleft palate (*) and a supernumerary deciduous tooth (arrow)

A 3.970 kg (weight 94^th^ centile/ + 1.52 SDS; length 87^th^ centile; head circumference 57^th^ centile) first born female neonate was referred to our neonatal intensive care unit on day 1 of live with hypoglycemia (glycemia 2.11 mmol/L) and congenital syngnathia making oral feeding impossible. Her young parents were nonconsanguineous. Family health history and perinatal history were unremarkable, except for shoulder dystocia and nuchal cord. The maternal oral glucose tolerance test was normal, and no placental or amniotic fluid anomalies were detected. Apgar scores were 8 and 10 at one and five minutes, respectively. On examination the baby was showing macrosomy, overfolded superior helix ear malformation, broad nasal bridge, thin lips, mild retrognathia, numerous fibrous bands between mandibular and maxillary gingival arch and tongue-palate fusion (Fig. 1a-b) impeding the exploration of the oral cavity to ascertain the presence of a possible cleft palate. There were no lip abnormalities. She had measured clitoral hypertrophy, hypoplasia of the labia majora (Fig. 1c), bilateral cutaneous syndactyly of II and III toes, cutaneous syndactyly of IV and V right toes, and pyramidal skinfold overlying the nails of both halluces (Fig. 1d). Total parenteral nutrition was started. Diagnostic workup by means of clinical, radiological and ultrasound (US) investigations revealed other comorbidities. Awake nasopharyngoscopy evidenced palatal hypermobility as a suspicious sign of (submucosal) cleft palate; however, the laryngeal structures and laryngeal air column were normal. Three-dimensional imaging with multidetector spiral computed tomography virtually reconstructed the facial skeleton and revealed cleft palate, underdeveloped mandibular condyles, reduced temporomandibular joint spaces and flattening of its bony components, as well as a supernumerary deciduous maxillary premolar, but no synostosis (Fig. 2a-b). Cardiac and transfontanellar cerebral US and fundoscopy were normal.

All treatment options were discussed with parents. At 6 days of life, surgery was performed under analgosedation with preserved spontaneous breathing. Synechiae of the anterior oral cavity were ablated by electrosurgery. On the right side the soft-tissue fusion of the tongue base with the uvula (glossopalatal ankylosis) was released and a cleft palate was discovered. Passive mouth opening and tongue protrusion achieved after the release were quite satisfying. Subsequent laryngoscopic exploration of pharyngeal and laryngeal structures were normal.

Due to still restricted mouth opening, enteral nutrition by Haberman feeder was encouraged shortly after surgery and assisted by intensive logopedic therapy gymnastics but there was a severe incoordination of sucking and swallowing. Therefore, she was nourished mainly by a nasogastric tube. At one month of age, the parents agreed to perform a percutaneous endoscopic gastrostomy tube placement and the infant was discharged home three days later. Afterwards it was replaced by a low-profile button. At two month, more extensive ocular and auditory electrophysiological studies and magnetic resonance neuroimaging resulted normal. At seven months of age, a primary palatoplasty and a surgical separation of webbed toes were performed in one operating session. The infant underwent a palatal revision surgery aged nine months. In consideration of the external genital anomalies, US and voiding cystourethrography studies did not reveal other associated genitourinary malformations. By continuing the physiokinesitherapy, child’s growth, neuromotor and cognitive development, general and oral health are regular at age 5 years.

During neonatal hospitalization, karyotyping was performed and resulted normal (46,XX), while the array-based comparative whole-genome hybridization (aCGH) using the Agilent® 8 × 60 K microarray detected a 527 kb interstitial paternal duplication of 7q36.3(158591696_159118566) and a 122 kb maternal duplication of Xp11.22(53479677_53602100) encompassing respectively genes *WDR60* (OMIM *615,462), *VIPR2* (OMIM *601,970), *LINC00689*, *ESYT2* (OMIM *616,691) and genes *MIR98* (OMIM *300,810), *MIRLET7F2* (OMIM *300,721), *HUWE1* (OMIM *300,697). Parental testing confirmed the paternal and maternal origin of each duplication, respectively.

Successively, a targeted gene panel sequencing (480 genes for orofacial clefts) revealed a novel *de novo* heterozygous c.262A > G mutation in exon 4 of *IRF6* gene (OMIM *607,199) causing an asparagine to aspartate missense mutation (p.Asn88Asp) that was compatible with the phenotype.

## Discussion and conclusion

We have reported on a female patient with oromaxillofacial abnormities (synechiae, cleft palate, craniofacial dysmorphisms, dental anomaly) and extraoral malformations (skinfold overlying the nails of both halluces, syndactyly, abnormal external genitalia) as phenotypical cardinal signs of autosomal dominant IRF6-related disorders clinically known as (Demarquay-)van der Woude syndrome 1 (VWS1; OMIM #119,300) and/or popliteal pterygium syndrome (PPS; OMIM #119,500). However, suggestive findings as the pathognomonic lower lip pits in 88% of VWS patients [4] and the eponymous popliteal pterygia in 97% of PPS patients [5] were absent in our patient. Oral clefts are the most frequent congenital anomalies reaching a birth prevalence about 1:660 in Europe (Eurocat data). An IRF6-related disorder resembles the most common single gene cause of oral clefts. In Italy, the prevalence is 3% in all cases of cleft palates [6], while in familiar oral clefts the frequency is increasing to 16% in the European area [7].

Congenital syngnathia is a rare anomaly which presents as fusion of the jaws with inability to open the mouth at the time of birth. It can have different degrees of severity in maxillofacial complex, ranging from mucosal bands to bony fusions involving maxilla, mandible, temporomandibular joint, and even zygoma. Oral synechiae are further classified as primary due to persistent buccopharyngeal membrane or secondary to formation of ectopic membranes as in our case [8]. We have found that, even after a timely release, severe feeding difficulties due to unused fetal swallowing may persist beyond the neonatal age. If the treatment is delayed, underdevelopment of mandible as well as temporomandibular joint disorders and oral-motor dysfunctions may deteriorate significantly [3]. On the other hand, bypassing oral nutrition by nasogastric tube or even by gastrostomy without release of syngnathia is dangerous, due to the impossibility to intervening in case of aspiration of gastric contents and the impossibility to detect possible associated malformations like oral clefts, smaller lower pharyngeal airways [9], tracheoesophageal fistulae or severe gastrointestinal anomalies, among others [8, 10, 11]. However, in rare mild cases the synechiae have been preserved for use as a flap during surgical palatal closure [12], also because there are scarring problems in some patients and other palatal reconstruction techniques may need revisions [13, 14]. IRF6 as a transcription factor regulates keratinocyte proliferation, granulation tissue maturation, and cytokine levels, thereby having a strong implication in wound healing [14].

Finding both cleft palate and congenital intraoral synechiae reduces the risk from more than 500 possible involved genes in orofacial clefts to about 20. This clinical association could be the phenotype of cleft palate lateral synechiae syndrome (CPLS or cleft palate and congenital alveolar synechia syndrome) [7, 12], orofaciodigital syndromes (OFD), Fryns syndrome [15] and IRF6-related disorders or other syndromes interacting with IRF6 in a reciprocal complex network [16]. Actually, it is debated if the first genetic approach should be a direct Sanger sequencing of only *IRF6*, a panel gene study of more genes, and/or an aCGH [7, 17]. Indeed, overlapping features, sometimes including neonatal hypoglycemia, warrant a more general investigation, since there are other genes of interest in the differential diagnosis of IRF6-related disorders. These should at least include: *BMP4* (Orofacial cleft 11; OMIM #600,625), *CDH7* (CHARGE syndrome; OMIM #214,800), *FGF8* (hypogonadotropic hypogonadism 6; OMIM #612,702), *FGFR1* (FGFR1-related hypogonadotropic hypogonadism; OMIM #147,950), *GRHL3* (VWS2; OMIM #606,713), *KDF1* (Ectodermal dysplasia 12; OMIM #617,337), *KMT2D*/*KDM6A* (Kabuki syndrome; OMIM #147,920/#300,867), *MSX1 (*MSX1-Related Disorders like Ectodermal dysplasia 3; OMIM #189,500), *OFD1* (OFD1; OMIM #311,200), *RIPK4/CHUK* (Bartsocas-Papas syndrome; OMIM #263,650/#619,339), *TFAP2A* (branchio-oculo-facial syndrome; OMIM #113,620), and *TP63* (TP63-related disorders like Hay-Wells syndrome; OMIM #603,273) [16, 18, 19]. Interestingly, it has been reported an Italian girl with cooccurrence of clinical diagnosed Kabuki syndrome and VWS [20]. We did not consider Beckwith-Wiedemann syndrome since the patient did not meet the criteria for molecular testing despite transient hypoglycemia encountered only once at admission and the birthweight + 1.52 SDS above the mean (not > 2 SDS).

We found a *novel* mutation (p.Asn88Asp) in the DNA binding domain of *IRF6*. This variant has never been reported in the literature and in dedicated databases (HGMD Professional, OMIM, ClinVar). According to the American College of Medical Genetics and Genomics (ACMG) and Association for Molecular Pathology (AMP) combining criteria [21], this novel variant results pathogenic satisfying the following evidences. First, this *de novo* variant is present only in the propositus and not in her parents and there is no family history of disease, classifying the variant as strong pathogenic (PS2). Second, the allele frequency has not been recorded in the Genome Aggregation Database (www.gnomad.broadinstitute.org) (moderate pathogenic 2 – PM2). Additional computational (in silico) and predictive data interpretation predicted a deleterious effect on the gene product in a phylogenetic conserved sequence (pathogenic supporting 3 – PP3; tools for mutational analyses used: PolyPhen-2 = 1.00/1.00, SIFT = 0.00/0.00, MutationTaster = 1.00/1.00, CADD PHRED = 29/20, MutationAssessor = 3.46/5.00, phyloP-Vertebrate = 4.75/6.42, phyloP-Primate = 0.53/0.65, PhastCons = 1.00/1.00). Finally, other similar asparagine missense mutations as asparagine to histidine (p.Asn88His), tyrosine (p.Asn88Tyr), serine (p.Asn88Ser) or isoleucine (p.Asn88Ile) mutations have been reported in VWS1 patients of North European or Latin American origin (PM5) [5, 22, 23], further suggesting that this is an important conserved residue. It is one of the 17 amino acids that are highly predicted to contact the DNA in the 120 amino acids DNA binding domain [5, 24, 25]. Asparagine as a polar amino acid with no charge on the “R” group (side chain) is taking part in the hydrogen bond formation in protein molecules. On the contrary, aspartate has a negatively charged “R” group becoming more acidic and having H-bond acceptors but not donors impeding hydrogen bonding. Thus, this makes plausible that this amino acid change may alter *IRF6* gene function, likely through reduced DNA-binding ability. The resulting phenotype is compatible with VWS1 as well as PPS, consistent with previous reports of diverse expression of a unique mutation within a family due to epigenetic processing [5, 26, 27]. Interestingly, in our patient we have confirmed the normal presence of both chromosomes 1 by aCGH but detected two additional duplications in 7q36.3 and Xp11.22 of paternal and maternal origin, respectively. Both parents do not show any pathologic phenotype, attributing an uncertain significance of these duplications. However, the Xp11.22 variant involving two microRNA genes (*MIR98* and *MIRLET7F2*) could be worth to more detailed studies in view of a possible epigenetic effect. The let-7 microRNA family has been reported to orchestrate a regulome of cell proliferation and differentiation in cranial neural crest cells and lip mesenchymal cells and can modify craniofacial embryology [28].

During genetic counseling the parents have been informed about prenatal diagnosis options due to the estimated recurrence risk of 0.76% (95%-CI: 0.50–1.16; calculated according to formula by Jónsson H et al. 2018) considering the theoretically possible parental germline mosaicism of mutation [29].

Our reported case carrying a novel mutation can contribute to expand understandings of molecular mechanisms underlying synechia and orofacial clefting and to correct diagnosing of incomplete or overlapping features in IRF6-related disorders.

Thus, additional multidisciplinary evaluations to establish the phenotypical extent of the IRF6-related disorder and to address family counseling should not only be focused on the surgical corrections of syngnathia and cleft palate, but also involve comprehensive otolaryngologic, audiologic, logopedic, dental, orthopedic, urological, and psychological evaluations.

## Data Availability

The datasets used and/or analyzed during the current report are available from the corresponding author on reasonable request.

## Abbreviations

aCGH: Array-based comparative whole-genome hybridization
*BMP4*: Bone morphogenetic protein 4
*CHUK*: Component of nuclear factor kappa-B kinase complex
*CDH7*: Cadherin 7
*ESYT2*: Extended synaptotagmin-like protein 2
*FGF8*: Fibroblast growth factor 8
*FGFR1*: Fibroblast growth factor receptor 1
*GRHL3*: Grainyhead-like 3
*HUWE1*: HECT domain-containing ubiquitin E3 ligase 1
*IRF6*: Interferon regulatory factor 6
*KDF1*: Keratinocyte differentiation factor 1
*KDM6A*: Lysine demethylase 6A
*KMT2D*: Lysine-specific methyltransferase 2D
*LINC00689*: Long Intergenic Non-Protein Coding RNA 689
*MIRLET7F2*: Micro RNA Let-7f-2
*MIR98*: Micro RNA 98
*MSX1*: MSH homeobox 1
OFD: Orofaciodigital syndrome
PM2: Moderate pathogenic type 2
PP3: Supporting pathogenic 3
PPS: Popliteal pterygium syndrome
PS2: Strong pathogenic type 2
*RIPK4*: Receptor interacting serine-threonine kinase 4
*TFAP2A*: Transcription factor AP2-alpha
*TP63*: Tumor protein p63
US: Ultrasound
*VIPR2*: Vasoactive intestinal peptide receptor 2
VWS1: Van der Woude syndrome
*WDR60*: WD repeat-containing protein 60
